# Involvement of Cytokines in the Pathogenesis of Salt and Water Imbalance in Congestive Heart Failure

**DOI:** 10.3389/fimmu.2017.00716

**Published:** 2017-06-19

**Authors:** Zaher S. Azzam, Safa Kinaneh, Fadel Bahouth, Reem Ismael-Badarneh, Emad Khoury, Zaid Abassi

**Affiliations:** ^1^Department of Physiology and Biophysics, Technion, Israel Institute of Technology, Haifa, Israel; ^2^Internal Medicine “B”, Rambam Health Care Campus, Haifa, Israel

**Keywords:** heart failure, alveolar epithelium, renal cells, inflammation, cytokines, alveolar fluid clearance

## Abstract

Congestive heart failure (CHF) has become a major medical problem in the western world with high morbidity and mortality rates. CHF adversely affects several systems, mainly the kidneys and the lungs. While the involvement of the renin–angiotensin–aldosterone system and the sympathetic nervous system in the progression of cardiovascular, pulmonary, and renal dysfunction in experimental and clinical CHF is well established, the importance of pro-inflammatory mediators in the pathogenesis of this clinical setting is still evolving. In this context, CHF is associated with overexpression of pro-inflammatory cytokines, such as tumor necrosis factor-α, interleukin (IL)-1, and IL-6, which are activated in response to environmental injury. This family of cytokines has been implicated in the deterioration of CHF, where it plays an important role in initiating and integrating homeostatic responses both at the myocardium and circulatory levels. We and others showed that angiotensin II decreased the ability of the lungs to clear edema and enhanced the fibrosis process *via* phosphorylation of the mitogen-activated protein kinases p38 and p42/44, which are generally involved in cellular responses to pro-inflammatory cytokines. Literature data also indicate the involvement of these effectors in modulating ion channel activity. It has been reported that in heart failure due to mitral stenosis; there were varying degrees of vascular and other associated parenchymal changes such as edema and fibrosis. In this review, we will discuss the effects of cytokines and other inflammatory mediators on the kidneys and the lungs in heart failure; especially their role in renal and alveolar ion channels activity and fluid balance.

## Introduction

Congestive heart failure (CHF) has recently become a major medical problem in the developed countries with increased rates of mortality and morbidity, particularly among the elderly population. CHF constitutes an enormous economic burden on health service because of the expensive costs of the various therapeutic modalities, frequent hospital admissions, and poor quality of life. In the developed countries, it is estimated that up to 2% of the adult population suffers from this syndrome; whereas, in patients ≥65 years of age, the prevalence surges to more than 10% (1). The pathophysiologic conditions of CHF are various and include either decreased cardiac output due to loss of cardiac muscle tissue as it is observed in myocardial infarction, myocarditis and dilated cardiomyopathy; or increased filling pressures of the heart as it is evident in hypertension, hypertrophic and restrictive cardiomyopathies, and certain valvular diseases. CHF can also develop due to a volume overload deriving from arteriovenous shunts or fistulas and administration of fluid excess.

Understanding the underlying mechanisms leading to the development of CHF and its complications is therefore essential for optimizing the treatment of CHF and exploring novel therapies that aim to improve the outcome of the disease (2). Since the early 1980s, the importance of vasoconstrictor neurohormonal systems in the pathogenesis of CHF has been increasingly recognized. Numerous studies in patients and in experimental models of CHF have established the important role of the renin–angiotensin–aldosterone system (RAAS) and the sympathetic nervous system (SNS) in the progression of cardiovascular and renal dysfunction in CHF. It is now accepted that excessive neurohormonal activation may adversely affect cardiac function and the hemodynamic condition by enhancement of systemic vasoconstriction and promoting salt and water retention by the kidney. In addition, prolonged activation of the SNS and RAAS may have direct deleterious actions on the myocardium, independent of their systemic hemodynamic effects (3–5). However, generally, inflammation plays an important role in most cardiac diseases, and receptor-mediated innate immunity is primarily investigated with respect to toll-like receptors. However, the role of the innate immune system in heart failure has been controversial (6).

Cytokines that are composed of a vast array of relatively low molecular weight, pharmacologically active proteins; have been implicated in the progression of CHF. The most important cytokines are tumor necrosis factor-α (TNF-α), interleukin (IL) 1β, and IL-6. These cytokines share some of their major characteristics (redundancy), and all act in a pro-inflammatory sense (7). Adhesion molecules, autoantibodies, nitric oxide (NO), and endothelin-1 are also thought to be relevant to the pathogenesis of CHF (8).

Recently, it was shown in patients with acute decompensated heart failure (ADHF) that following standard treatment of ADHF, the monocyte profile and circulating inflammatory markers (C-reactive protein and IL-6) shifts to more closely resemble those of healthy controls, suggesting the contribution of systemic inflammation to the pathophysiology of ADHF (9). We and others have shown the deleterious consequences of heart failure on the lungs and the kidneys; therefore, we decided in this review to focus on the effects of cytokines and other inflammatory mediators on the lungs and the kidneys in heart failure; especially their role in renal and alveolar ion channels activity and fluid balance.

## The Contribution of the Immune System to Heart Failure

There are several theories regarding the activation of the immune system in heart failure (10). One hypothesis is based on the consequences of heart failure, that is, systemic venous congestion including the mesenteric venous system with consequent bowel edema and increased permeability that leads to bacterial translocation, endotoxin release and resultant activation of the immune system (11). The second theory is related to the ability of the failing heart to produce cytokines; Torre-Amione et al. have shown that TNF-α mRNA and TNF-α protein were present in the explanted hearts from dilated cardiomyopathy and ischemic heart disease patients but not in non-failing hearts (12).

In the third hypothesis, the state decreased cardiac output in heart failure causes systemic tissue hypoxia with subsequent systemic inflammation, which in turn may be the primary stimulus for increased TNF-α production (13).

The heart undergoes extensive structural and functional remodeling in response to injury, central to which is the hypertrophy of cardiac myocytes, with excessive deposition of extracellular matrix (14). Myocardial fibrosis is commonly categorized as one of two types: reactive fibrosis or replacement fibrosis. Reactive fibrosis occurs in perivascular spaces and corresponds to similar fibrogenic responses in other tissues; replacement fibrosis occurs at the site of myocyte loss.

Myocardial fibrosis is attributed to cardiac fibroblasts, which resides in the myocardium and is confirmed to be abundant (15). Following myocardial injury, all types of fibroblasts proliferate and differentiate into myofibroblasts, a process that is orchestrated by classic mediators such as TGF-β1, endothelin-1, and angiotensin II (Ang II). Notably, fibrosis is accelerated as result of intercellular interaction and cross talk; in this case, between activated fibroblasts and cardiomyocytes (16).

The effects of fibrosis on the heart muscle are various and include impairment of cardiac function, both systolic and diastolic. It also caused electrical instability and the development of fatal ventricular arrhythmias. This arrhythmogenic activity occurs in areas that couple fibroblasts and cardiomyocytes due to discontinuous slowing of conduction and consequent arrhythmia (17).

The CORONA study that included 1,464 patients with chronic ischemic systolic HF demonstrated that serum levels of TNF-α, soluble TNF receptors type I and II (sTNF-RI and sTNF-RII), and the chemokines monocyte chemoattractant protein-1 and interleukin-8 (IL-8) were independent predictors of all endpoints (all-cause mortality, cardiovascular mortality, and worsening heart failure). After further adjustment for estimated glomerular filtration rate (GFR), the ApoB/ApoA-1 ratio, NT-proBNP, and high-sensitivity C-reactive protein, only IL-8 remained a significant predictor of all endpoints (except the coronary endpoint), while sTNF-RI remained independently associated with CV mortality (18). Recently, in concordance of this study, it was reported IL-8 was negatively correlated with the left ventricular end-diastolic diameter and positively with left ventricular systolic volume (19). However, it should be emphasized that the elevated levels of cytokines in general and in heart failure, in particular, may not be responsible for tissue injury, rather it may reflect a concomitant phenomenon where cytokines could be used as biomarkers for heart failure but not effectors.

### Pulmonary System

The alveoli are composed of thin layer of epithelial cells; alveolar epithelial cells type I and type II (AECI and AECII, respectively) that occupy together 99% of surface area of the lungs and play a crucial role in breathing and preserving lung homeostasis. There are also alveolar residential macrophages that protect the lungs from pathogens and regulate lung immune response (20).

Alveolar macrophages—AMφ comprise 95% of bronchoalveolar lavage and are part of cellular compartment of innate immunity that has an essential role in pathogen defense. Another type of macrophages is interstitial macrophages or bone-marrow derived macrophages that are also involved in the process of lung defense (21).

#### Alveolar Fluid Clearance (AFC)

**Figure 1:**
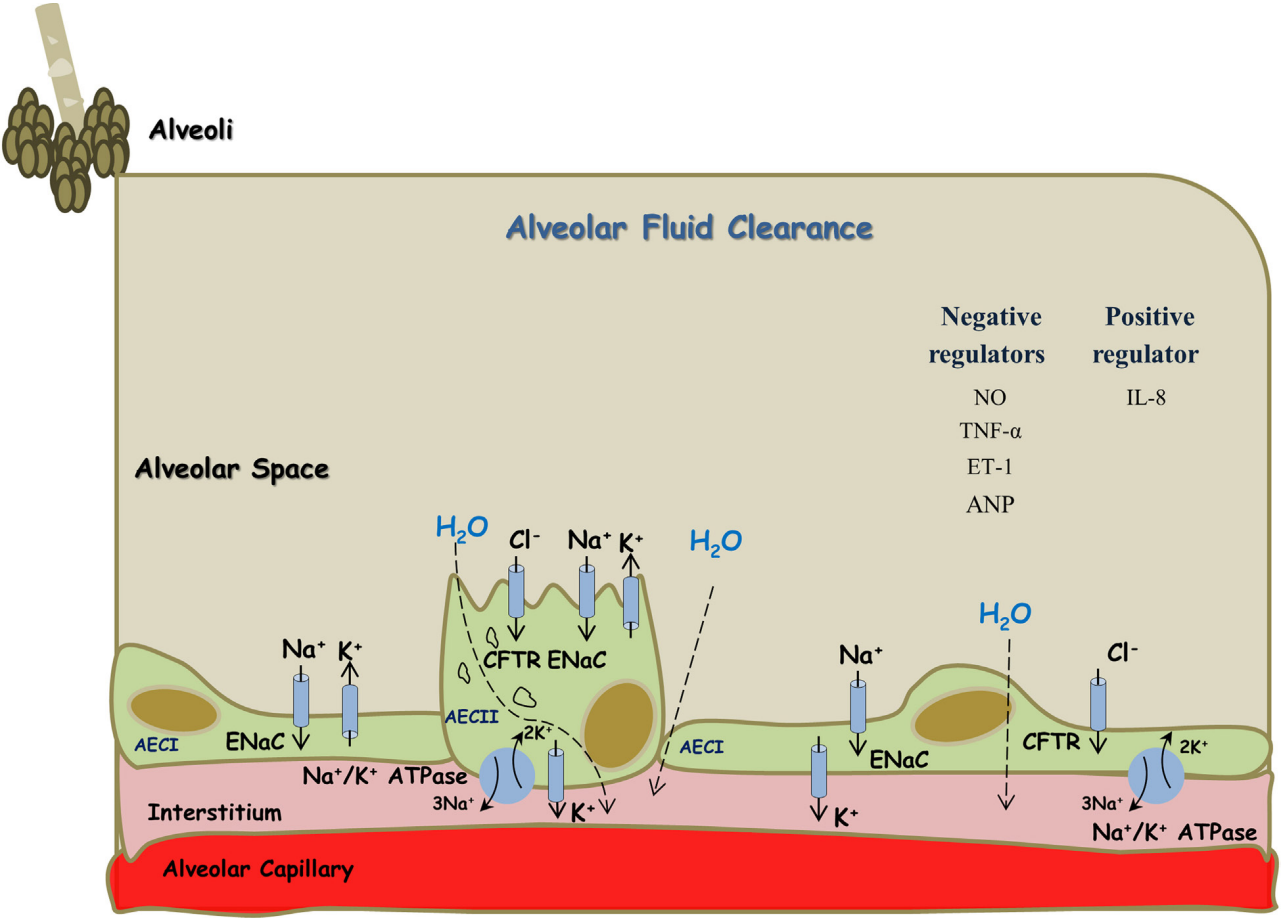
Alveolar fluid clearance process in the lung epithelium. Sodium is actively transported from alveolar space to the lungs’ interstitium and blood vessels; achieved mainly by apical ENaC and basolateral Na^+^/K^+^ ATPase located at AECI and AECII. This results in the formation of osmotic gradient, which drives transcellular and paracellular movements of water molecules. Some regulators, including cytokines, negatively affect this process while others appear to be with positive effects. AECI, alveolar epithelial cells type I; AECII, alveolar epithelial cells type II.

Active sodium (Na^+^) transport across the alveolar-capillary barrier is important in keeping the airspaces free of fluid in healthy conditions and for the resorption of lung edema in pathologic conditions. Briefly, Na^+^ enters the alveolar epithelial cells through apical amiloride sensitive Na^+^ channels (ENaC), and by a process that consumes energy is pumped out of the cell by the Na,K-ATPase located in the basolateral membrane in exchange for potassium entry on a ratio of 3:2 Na^+^–K^+^ against their chemical gradient (20, 22–27) (Figure 1). ENaC constitutes the rate limiting step for sodium absorption in epithelial cells of various sites including distal renal tubule, distal colon, exocrine glands, and lungs. Concerning the latter, ENaC plays a critical role in AFC. A support for this notion was derived from Hummler et al. who demonstrated that AFC in knockout mice to ENaC was severely attenuated with resultant fatal respiratory distress (27). Notably, non-selective Na^+^ channels (NSC) and cyclic nucleotide-gated channel have been shown to be involved in the process of AFC, however to a lesser extent than ENaC (28). In addition, K^+^ ions are recycled by basolateral K^+^ channels, which also participate in the control of Na^+^ and fluid absorption (26). It has been shown that AFC is modulated by several pharmacologic modalities and interventions; such as catecholamines, angiotensin, vasopressin, endothelin, gene therapy, hypercapnia, hyperoxia, sepsis, and others (20, 29–33). Notably, Ang II decreased AFC *via* c-AMP–Na, K-ATPase pathway. Whereas, it was reported that Ang II plays a role in lung fibrosis by phosphorylating p38 and p42/44 kinases (also called extracellular signal-regulated protein kinases, ERK 1/2) (31). Ang II-induced mitogen-activated protein kinase (MAPK) activation has been implicated in myocardial hypertrophy, inflammation and neurotransmitter catecholamine synthesis, and release in the brain (34–36). These two kinases play a distinct role in the induction and signaling of pro-inflammatory cytokines. Specifically, fibroblasts stimulated with Ang II showed a strong time-dependent expression of COX-2 protein. The p38 MAPK inhibitor SB203580 but not the p42/44 MAPK-inhibitor PD98059 suppressed Ang II-induced COX-2 protein expression, a pro inflammatory enzyme (37). Likewise, blockade of Ang II receptors type I and II (AT1 and AT2, respectively) reduced the levels of TNF-α and its damage on renal tubular cell injury, thus exerting cytoprotective effects (38). Concerning the interaction between the RAAS and CNS systems, Wei et al. demonstrated that Ang II stimulates MAPK to upregulate brain AT1 receptors in rats with HF (39). Similarly, these authors demonstrated that Ang II-activated MAPK signaling pathways contribute to sympathetic excitation in HF (40). Specifically, intracerebroventricular administration of two selective p44/42 MAPK inhibitors, PD98059 and UO126, induced significant decreases in mean arterial pressure, heart rate, and renal sympathetic nerve activity in rats with HF but did not affect these parameters in sham controls. In addition, MAPK can be activated by other factors, such as pro-inflammatory cytokines and reactive oxygen species (41, 42), which are known to increase during inflammatory, pulmonary, and cardiac diseases. ERK1 and ERK2 play a crucial role in the pathogenesis of cardiac and vascular diseases. In this context, it was found that ERK1/2 and p38 MAPK activation occurred within 10 min of transverse aortic constriction, a model of pressure load heart failure (43). Similarly, activation of ERK, Jun kinase (JNK), and p38 MAPK has been demonstrated in other clinical and experimental heart failure (44).

The ability of the lungs to clear edema is impaired in acutely increased left atrial pressure (45–48). The underlying mechanisms are not fully understood; it has been assumed that NO synthesized in the alveolar endothelial cells attenuated the ability of the lungs to clear fluids *via* alveolar endothelial–epithelial interactions (45). The addition of Ang II to cultured vascular smooth muscle cells did not induce neither nuclear factor kappa B (NF-κB) activation nor iNOS or VCAM-1 expression. However, when added together with IL-1β, Ang II, through activation of the (AT1) receptor, inhibited iNOS expression and enhanced VCAM-1 expression induced by the cytokine. The inhibitory effect of Ang II on iNOS expression was associated with a downregulation of the sustained activation of extracellular signal-regulated kinase (ERK) and NF-κB by IL-1β, whereas the effect on VCAM-1 was independent of ERK activation. The effect of Ang II on iNOS was abolished by inhibition of p38 MAPK with SB203580. The authors concluded that Ang II, by a mechanism that involves p38 MAPK, differentially regulates the expression of NF-κB-dependent genes in response to IL-1β stimulation by controlling the duration of activation of ERK and NF-κB (49).

In chronic heart failure, however, the ability of the lungs to clear edema is increased particularly in compensated CHF (50, 51). Verghese et al. have shown that in most of the patients with hydrostatic pulmonary edema, AFC is intact or even increased. Notably, in this population, there was a trend though insignificant toward better outcomes (52).

De Vito reported that Na^+^/H^+^ Exchanger isoform 1 might be a possible mediator of immunity involved in cytoplasmic pH (pH_i_) homeostasis and expression of cytokines and chemokines (53). Our laboratory is currently investigating the expression pattern of Na^+^/H^+^ Exchanger (NHE) isoforms in alveolar epithelial cells and to evaluate their involvement in AFC process in both control and heart failure rats. CHF was induced by the placement of arteriovenous fistula between the abdominal aorta and vena cava (50). Notably, one should bear in mind that many of the immune cell functions are coupled with pH_i_ modification. Specifically, an increase in pH_i_ represents an important signal for cytokine and chemokine release, whereas a decrease in phagosomal pH can induce an efficient antigen presentation (53). Thus, our hypothesis in this regard speculates a potential role of one of the NHE isoforms in the inflammatory aspect of heart failure in general and pulmonary system in particular.

##### The Effects of Cytokines on AFC

The effects of cytokines on AFC were examined on a variety of acute lung injury (ALI) models and found to play a controversial role (18, 19, 54).

The role of the immune system in patients with acute respiratory distress syndrome (ARDS) and ALI is well known; briefly, soon after lung injury, endothelial cells are damaged with gap formation that allows fluid permeability, activation, and migration of neutrophils with activation of pro-inflammatory cytokines such as TNF-α, IL-1β, and the transcriptional regulatory NF-κB. Notably, in response to stimuli, such as infection, NF-κB is activated with consequent cellular responses that lead to pulmonary edema due to ALI/ARDS (55). Peteranderl et al. recently demonstrated that in mice lungs infected with influenza A (IAV), the rate of AFC was decreased *via* inhibiting the recruitment of Na,K-ATPase α subunit to the plasma membrane (54). It was demonstrated that this process was mediated by a paracrine cross talk between the infected and non-infected AEC and alveolar macrophages. The mediators that were involved in this interaction were principally interferon α and to lesser degree IFNβ and an IFN-dependent elevation of macrophage TNF-related apoptosis-inducing ligand. Interestingly, interruption of this cellular cross talk accelerates the rate edema resolution, which is of biologic and clinical importance to patients with IAV-induced lung injury (56).

TNF-α levels are known to be increased in heart failure. It was shown that LV ejection fraction was depressed in transgenic mice overexpressing TNF-α in cardiomyocytes; this effect was dependent on TNF-α gene dosage (57). However, the knowledge regarding the role of cytokines on AFC in the context of heart failure is limited. Rezaiguia et al. have shown that TNF-α instilled in normal rats increased alveolar liquid clearance by 43% over 1 h compared with control rats; conceivably, due to ENaC stimulation. TNF-α, which is secreted from alveolar macrophages binds to TNF receptors located on alveolar epithelial cells, where it induces its effects probably *via* upregulating of G proteins coupled ENaC. This effect is mediated *via* the lectin-like domain of TNF-α (58, 59). Another suggested mechanism is recruitment of ion channels to the cell membranes (60, 61). Moreover, it was demonstrated that in a model of ischemia–reperfusion in rats; AFC was upregulated, at least partly *via* TNF-α-dependent mechanism (62). On the other hand, it was reported that treating alveolar epithelial cells with TNF-α, the mRNA expression of ENaC subunits was decreased with compatible decrease in activity (63, 64). Therefore, these studies demonstrated that exposure to TNFα decreases ENaC mRNA and protein expression, as well as ENaC function both in alveolar type II cells and in injured lungs.

In models of ALI, it was demonstrated that IL-8 mediated injury to both the endothelium and epithelium, with consequent high permeability edema formation and decreased AFC (54). In addition, pretreatment with anti-IL-8 antibodies successfully restored the rate of AFC to normal probably by attenuating injury to the epithelium (18, 19).

##### The Effect of NO and Endothelin on AFC

Endothelin-1 (ET-1), a potent vasoactive peptide produced by endothelial cells and released during injurious stimuli such as pulmonary hypertension and heart failure. It has been shown that elevated concentrations of ET-1 predict mortality and hospitalizations in HF patients (65). It is noteworthy that ET-1 has an inhibitory effect on lung edema clearance *via* an endothelial epithelial interaction. The underlying mechanism involves activation of endothelial ETB receptors and NO generation leading to alveolar epithelial Na,K-ATPase downregulation in a cyclic guanosine monophosphate (cGMP)-independent manner (32).

Kaestle et al. have explored the role of NO in both acute and chronic heart failure. They have shown that in isolated mouse lungs, hydrostatic edema formation was attenuated by NO synthase (NOS) inhibition. Similarly, edema formation was decreased in isolated mouse lungs of endothelial NOS-deficient mice. Whereas, in chronic heart failure model; AFC was preserved as a result of endothelial dysfunction and decreased NO generation. This effect is mediated by endothelial-derived NO acting as an intercompartmental signaling molecule at the alveolo-capillary barrier (45) (Figure 1).

### Renal System

**Figure 2:**
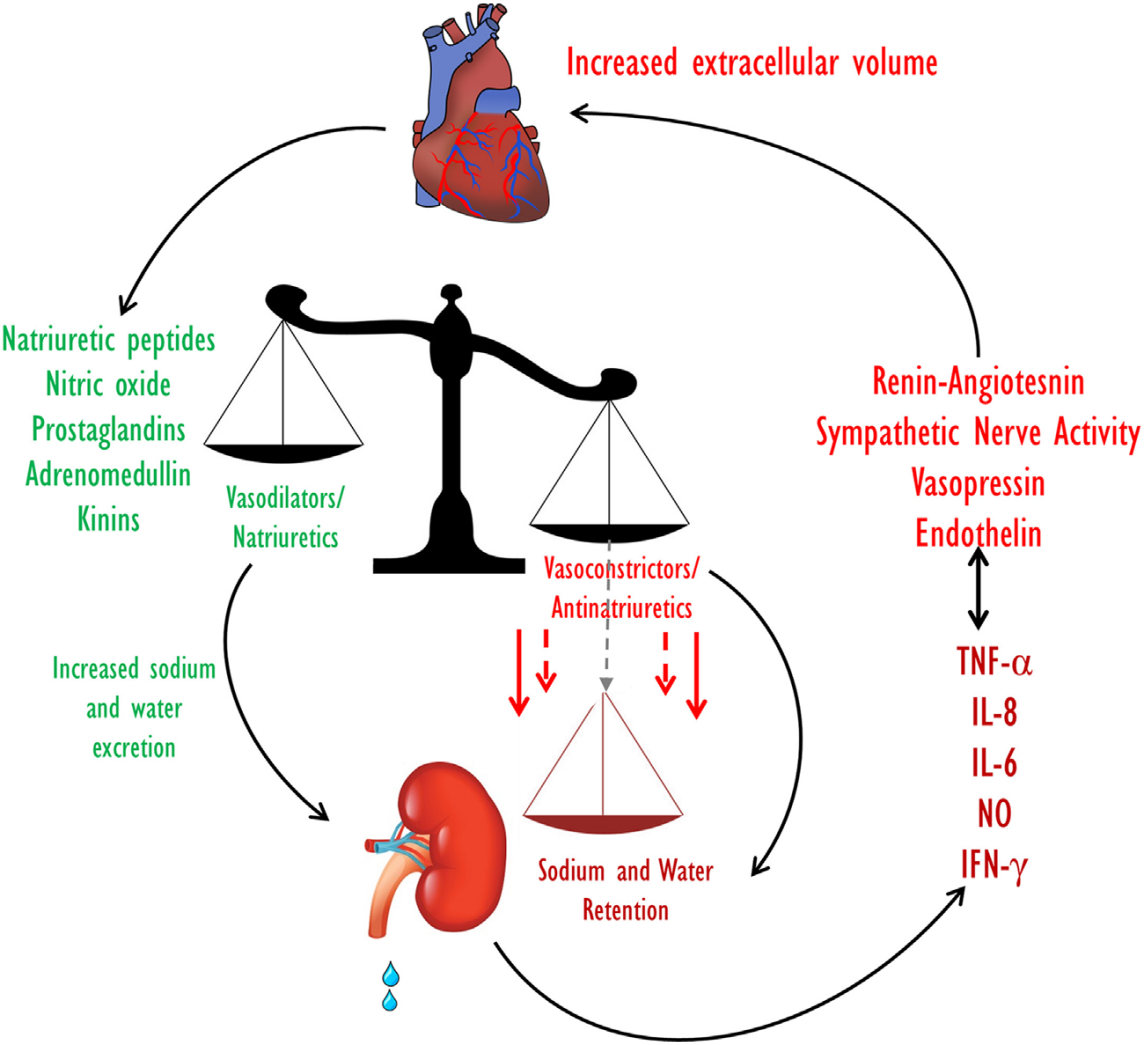
Extracellular fluid volume control in CHF. Volume homeostasis in CHF is determined by the balance between the natriuretic and the anti-natriuretic arms. In decompensated CHF, enhanced activities of the Na^+^-retaining systems along with activation of pro inflammatory substances overcome the effects of the vasodilatory/natriuretic systems, leading to a net reduction in Na^+^ excretion and eventually to an increase in ECF volume. CHF, congestive heart failure; ECF, extracellular fluid.

Kidney dysfunction is common in heart failure and is associated with an increased risk of mortality. The interaction between the heart and kidney in this setting is complex, involving multiple interdependent mechanisms including hemodynamic alterations and activation of multiple neurohormonal as well as pro-inflammatory systems (Figure 2).

#### Effects of Cytokines on Renal Handling of Water and Salt

The kidney is a major target organ of various hormones, and paracrine and autocrine substances. Many of the latter belong to the cytokines family, and some of the hormones that act on the kidney possess pro inflammatory properties. CHF is known to cause amplification of several pro-inflammatory mediators that can be detected at high concentrations in several vital organs and blood stream. The biologic sources of this chronic inflammatory state in CHF are not fully recognized. However, the heart and the kidneys produce a wide range of pro-inflammatory cytokines in response to activation of various neurohormonal systems and endotoxin accumulation as described below. Moreover, the hypo perfusion of the kidney during cardiorenal syndrome (CRS) results in sodium and water retention with further venous congestion. The biomechanical stretch of the vascular endothelium stimulates cytokine production (66).

##### The Renin–Angiotensin System

**Figure 3:**
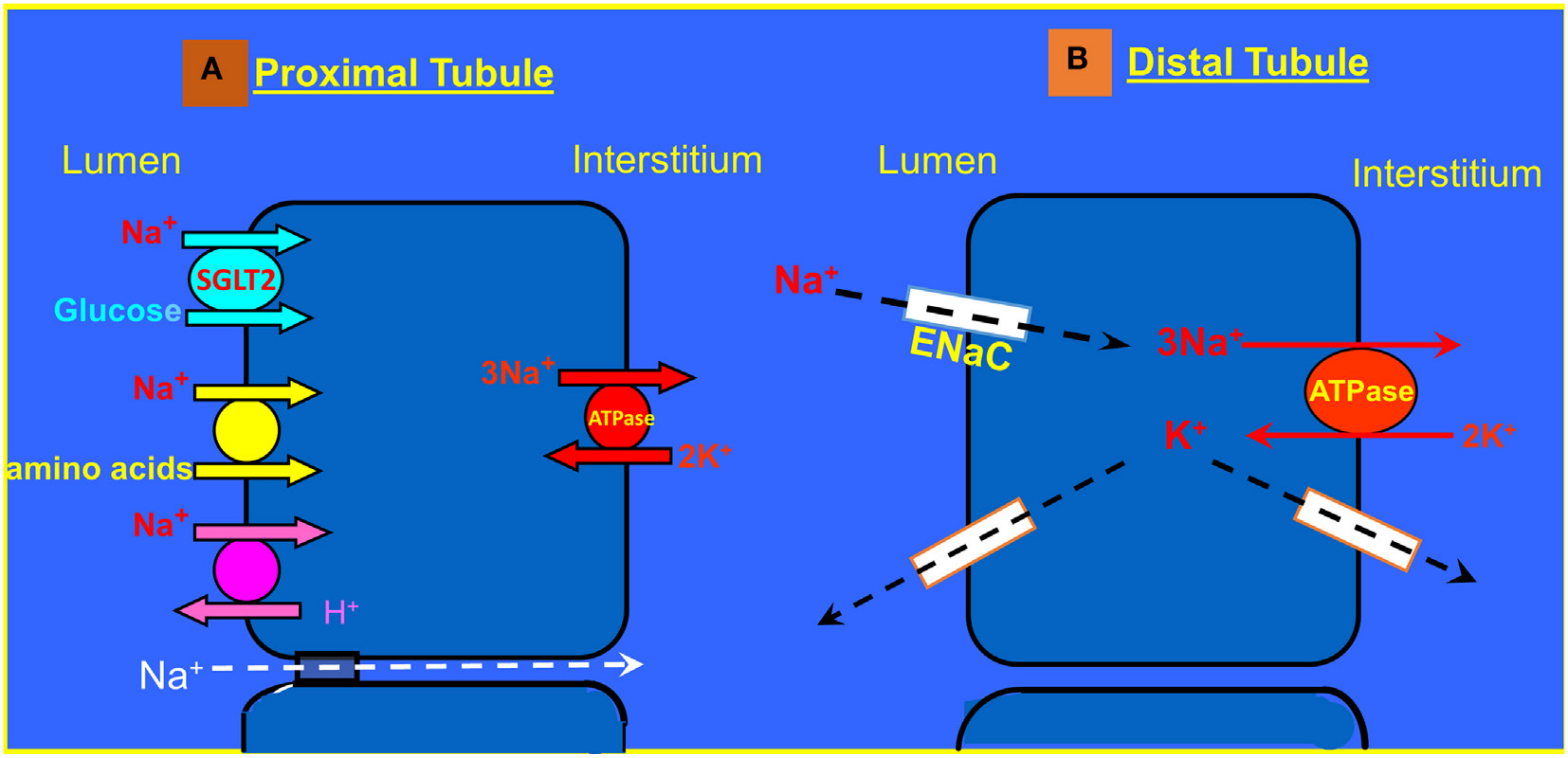
**(A)** Sodium transport in the proximal tubule. **(B)** Reabsorption of Na^+^ and K^+^ transport in the principle cells of the collecting duct.

The RAAS plays a major role in the pathogenesis of heart failure and responsible for the cardiovascular and renal manifestations of this disease (67–69). At the initial phase of CHF, the RAAS exerts beneficial effects aimed at BP maintenance by direct systemic vasoconstriction, or indirectly *via* augmentation of the SNS activity and by promoting renal sodium retention. However, as CHF progress, the biological activities of the RAAS turn to be deleterious and contribute significantly to the disease aggravation (67). The main active substances of the RAAS are Ang II and aldosterone, which play a key role in the adverse cardiac and renal manifestations of severe CHF (3). Concerning the kidney, both Ang II and aldosterone act directly on the proximal tubule and collecting dust where they enhance Na^+^ reabsorption *via* NHE3 and ENaC, respectively (Figure 3). Specifically, two-thirds of filtered sodium is reabsorbed in the proximal tubule *via* cotransporters along amino acid, glucose, phosphor as well as NHE3. Water follows sodium *via* aquaporin 1. At the distal tubule, sodium is reabsorbed by Na, K-cotransporter sensitive to thiazide. In the collecting ducts, a minimal amount of sodium (2–3%) is reabsorbed *via* amiloride sensitive ENaC that is upregulated by aldosterone. Water is reabsorbed in the collecting ducts *via* aquaporin 2 induced by vasopressin (70, 71). Furthermore, it is conceivable to reason that the anti-natriuretic effect of the RAAS is counterbalanced by the natriuretic/vasodilatory effect of atrial natriuretic peptide (ANP) on the kidney, thereby leading to substantial urinary retention of sodium and water retention with resultant edema formation (72, 73). In addition, increased activity of the RAAS contributes to the attenuated endothelial-dependent renal vasodilatation and the development of endothelial dysfunction characterizing CHF (74). Concerning the latter, it is attributed to several factors including the immune system. In support of this notion, it has been reported that T cells and various T cell-derived cytokines play a role in the pathogenesis of fluid/salt imbalance and elevated vascular resistance. For instance, various stimuli including Ang II, aldosterone, and catecholamines, which are known to be activated in CHF and hypertension increase the count of effector like T cells, which infiltrate the renal tissue in the perivascular regions of both arteries and arterioles (66, 75). There is also accumulation of monocyte/macrophages in these vascular beds (75). Both cell types release several cytokines including IL-17, IFN-γ, tumor necrosis factor-α (TNF-α), and IL-6, which cause renal damage and vascular dysfunction, resulting in avid sodium retention and elevated vascular resistance (75). By applying MI model induced by left coronary artery ligation in rats, Cho et al. demonstrated elevated activated monocytes (CC chemokine receptor 2^+^ ED-1^+^) in peripheral blood, along with the infiltration of ED-1^+^ macrophages and the increment of nuclear p65 in the kidney of MI rats, suggesting the contribution of NF-κB-mediated inflammation in the development of type I CRS. The inflammatory cytokines, IL-6, and tumor necrosis factor-α (TNF-α) mRNA expression, as well as microvascular endothelial permeability and tubular cell apoptosis, significantly enhanced in the kidneys of MI rats. These findings support the involvement of the immune activation/inflammation in the pathophysiology of CRS besides the hemodynamic alteration, pathological compensatory neurohormonal activation and oxidative stress (76).

As mentioned above, the RAAS activates the immune system but also the immune system activates certain components of RAAS (8). In this context, TNF-α and IL-6 stimulate the generation of angiotensinogen, exaggerate sodium retention and enhance renal fibrosis (77). One of the most famous representatives of the adverse cytokines in CHF is TNF-α, whose circulatory levels increase in correlation with the severity of the disease (66). In line with the deleterious pro-inflammatory role of Ang II in CHF, pharmacological blockade of the AT1 receptors in this clinical setting decreased the levels of pro-inflammatory cytokines including TNF-α (78, 79). Similarly, *in vivo* studies demonstrated that Ang II enhanced the expression of both TNF-α and IL-6 in the cardiomyocytes and in renal cortical and tubular cells (78, 80). These findings support the notion that the RAAS, especially Ang II, triggers the production of pro-inflammatory molecules in CHF.

Finally, several studies provided a keen linkage between oxidative stress and immune activation in several cardiovascular diseases including CHF and hypertension (81, 82). Interestingly, long-term activation of the RAAS impairs mitochondrial function, and increase oxidative stress burden which in turn can lead to renal injury and sodium and water retention (81). The major prooxidative stress stimulator is Ang II, where administration of the latter enhances renal mitochondrial oxidative stress and reduces GFR in rats with heart failure.

##### Sympathetic Nervous System

Activation of SNS is one of the hallmarks of CHF. It is well established the SNS mediates system and renal vasoconstrictor and salt retaining biological actions (83). However, experimental studies have demonstrated the involvement of pro-inflammatory cytokines TNF-α, IL-6, and IL-1β in CHF rats that were exposed to chronic stimulation of β-adrenergic stimulation with isoproterenol for 12 weeks as manifested by increased mRNA expression of these cytokines in cardiomyocytes and cardiac blood vessels. Nevertheless, the mRNA expression of NO was not increased. Thus, it is presumed that one mechanism underlying the beneficial effects of β-adrenergic blockade in heart failure may involve attenuation of TNF-α and IL-1β expression independent of iNOS and NO. In this context, β-blockade with metoprolol causes significant decline in TNF-α and IL-1β, but not IL-6 expression in the myocardium (84). It should be emphasized that the kidneys are preferentially innervate by sympathetic nerve fibers and the activity of renal sympathetic nerve is markedly increased in CHF. Stimulation of the renal nerve augments sodium reabsorption, decreases renal blood flow (RBF) *via* renal artery constriction, and stimulates renin release through β1 receptors on the juxtaglomerular apparatus (85).

Whether enhanced circulatory levels or locally produced cytokines in response to SNS or RAAS activation in CHF mediate some of the adverse renal actions of these systems remained largely to be elucidated. However, previous studies have shown that pro-inflammatory mediators such as TNF-α, IL-6, and CRP play a role in the pathophysiology of progressive renal injury and probably in salt and water imbalance characterizing various acute and chronic kidney diseases including CRS. As mentioned above, volume expansion associated with CHF promotes secretion of cytokines by endothelial cells. In this context, it was reported that TNF-α caused renal dysfunction as was evident by intravascular volume expansion due to salt and water retention (86). Likewise, oxidative stress enhances NaCl absorption by the thick ascending limb through activation of protein kinase C (87). Additional explanation to the avid sodium retention and water imbalance in heart failure is renal epithelial and endothelial damage, which leads to a loss of endo–epithelial barrier integrity and function. In this respect, Cho et al. have shown that CRS type I and II was associated with increased tubular damage marker as was evident by elevated levels of NGAL and tubular cell apoptosis. Moreover, macrophage infiltration and inflammatory cytokine expression possibly mediated by the NF-κB pathway, and microvascular endothelial damage increased significantly in kidneys at 3 days post-induction of MI, suggesting the important contribution of inflammation in the pathogenesis of type I CRS. These changes ultimately led to renal interstitial fibrosis along with chronically decreased heart function (type II CRS) (76). Although the direct effects of cytokines on ion transports in the kidney were no studied yet, the observation that heart failure is associated with enhanced renal TNF-α and IL-6 expression along tubular cell apoptosis supports such a role. In addition, microvascular endothelial injury characterized by endothelial cell apoptosis, alteration of actin cytoskeleton, or increased expression of leukocyte adhesion molecules has mediated the early phase of renal injury following ischemia and by facilitating leukocyte transmigration; it substantially contributes to tissue inflammation. In the long run, activation of the Ang II and SNS along renal hypoperfusion causes further activation of pro-inflammatory cytokines, which in their turn enhance neurohormonal activation thus creating a vicious cycle (88).

##### Endothelin

ET peptides are synthesized as preproETs in endothelium, heart, and kidney, processed into a big precursor and then converted into biologically active peptides such as ET-1 by 2 ET-converting-enzyme isoforms. Active ET-1 binds to ETA and ETB receptors (ETAR/ETBR) expressed in the kidney, lung, brain and cardiovascular system. ETAR activation generally causes vasoconstriction, while ETBR produces vasodilation. The status of the major components of the ET system in the kidney during CHF has been subject to intensive investigation. As outlined above, the endothelin system is over activated in CHF as evident by elevated levels of ET-1 in the circulation, cardiac and renal tissues as well as urinary excretion of this peptide (89–92). The deleterious and beneficial renal and cardiac ET effects are usually attributed to ETAR and ETBR, respectively (93, 94). However, ETAR blockade failed to improve cardiovascular outcomes and caused edema in clinical trials (95–98).

The heart and kidney are both important sources and key targets for ET. Cardiac myocytes and the renal glomerulus, vessels, and tubular epithelium each express ETA and ETB receptors (91). Elevated ET-1 levels correlate with CHF, hemodynamic dysfunction, and symptom severity (15, 99) and result in cardiac ETA upregulation along ETB downregulation (100–102). ET receptor antagonists attenuate experimental cardiac pathophysiology (103–107). ET-mediated renal pathology during CHF is an area of debate, but the potent reductions in RBF, GFR, natriuresis, and urine volume when ET-1 is increased to CHF levels supports a deleterious role for ET (108). ET levels also strongly correlate with renal dysfunction in patients with CRS (109, 110). However, a sustained cortical vasoconstriction and transient medullary vasodilation indicate renal responses to ET-1 are complex (108, 111). ET also produces dose-dependent changes in renal Na^+^ and water excretion, with high levels causing anti-natriuretic and antidiuretic effects, due to reduced GFR and RBF. In contrast, lower doses or local tubular *in situ* epithelial delivery produced ET-1 decrease tubular salt and water reabsorption, which are blocked by ETB antagonists (112).

Collectively, these findings suggest that ET signaling contributes to cardiac and renal dysfunction. Besides its endocrine/paracrine role in the regulation of the cardio vascular and renal hemodynamic and salt balance, ET-1 possesses pro-inflammatory and pro-fibrotic properties in pulmonary, cardiac, and renal diseases. Under these disease conditions, increases in ET-1 are critically involved in initiating and maintaining inflammation and injury, thereby promoting perturbations among the rest of salt and water balance. At the renal level, ET-1 stimulates the aggregation and accumulation of neutrophils, thus propagating glomerular inflammation, a process that can be inhibited by ETA receptor blockade (113).

##### Natriuretic Peptides (NPs)

This family consists of three members of the NPs: ANP, brain natriuretic peptide (BNP), and C-natriuretic peptides (114, 115). ANP and BNP are secreted mainly from the atria and ventricles, respectively, upon atrial distention and volume overload (116, 117). By binding to the NPR-A receptor, ANP and BNP induce the production of cGMP, which in turn promotes vasodilation, diuresis, natriuresis, and prevent cardiac remodeling, thus playing a major role in the homeostasis of blood pressure as well as of water and salt balance.

Interestingly, clinical and experimental heart failure are associated with high levels of circulating NPs, and today these peptides serve as biomarkers for HF. However, few studies have demonstrated that *de novo* synthesis of NPs in the renal tissue constitutes an essential pathway for maintaining normal blood pressure and fluid balance besides the NPs of cardiac origin. Ritter et al. were the first to report that primary cultures of neonatal and adult rat kidney cells produce and secrete ANP-like prohormone (118). Soon after, by using immunohistochemical staining, Greenwald et al. detected proANP predominantly in the distal cortical nephron (119). It was later that Ramirez et al. reported that all forms of proANP/ANP were found in the kidney, mainly in the proximal and distal nephron (120). ANP and their receptor, NPR-A, were highly expressed as well in these parts of the tubule. The natriuretic and diuretic effects of ANP are attributed to its stimulatory effect on GFR but also to its inhibitory action on ENaC in the collecting duct (121).

Interestingly, several studies demonstrated that ANP also act as autocrine/paracrine factor where it modulates various immune functions (122). There is keen evidence that ANP is locally produced by several immune cells, which also present specific natriuretic receptors. For instance, ANP stimulates the phagocytosis of macrophage and killing activity by ROS production, thus improving the innate immunity (123). Moreover, ANP inhibits lipopolysaccharide-induced NO release by macrophage cells and promotes the inactivation of NF-κB *via* cGMP (53, 123). In a recent study by Mitaka et al. (124), ANP pretreatment prevented kidney–lung cross talk in a rat model of renal ischemic reperfusion injury. Interestingly, this group has also shown that ANP posttreatment ameliorated injuries in kidney and lung by direct tissue protective effect and anti-inflammatory effects, which potentially inhibited interorgan cross talk (125). Zhu et al. have shown that ANP reduced the levels pro-inflammatory cytokines such as IL-1β, IL-6, IL-10, and TNF-α in rats with oleic acid-induced ALI (126). In agreement with its anti-inflammatory properties, ANP interferes with the expression of adhesion molecules such as ICAM-1 and E-selectin (66, 77).

Finally, in critically ill patients, BNP and NT-proBNP levels correlated with inflammatory markers such as CRP and leukocyte count (127). Likewise, patients with septic shock had elevated BNP concentration regardless of the presence of CHF condition. Although the involvement of these anti-inflammatory effects of NPs in the pathogenesis of renal function in CHF has not been completely understood, it may represent a counterbalance compensatory response to the activation of the adverse neurohormonal systems including RAAS, SNS, and ET-1.

##### NO System

Renal NO is a molecule synthesized from its precursor, l-arginine by the enzyme, NOS; this process takes place in several sites, mainly in the endothelial cells of the renal blood vessels but also in the tubular epithelial and mesangial cells. Notably, three different isoforms of NOS; NOS 1 (bNOS), NOS 2 (iNOS) and NOS 3 (eNOS). NO plays an important role in the regulation of renal hemodynamics and excretory function. Specifically, locally produced NO is involved in the regulation RPF, salt excretion, and renin release. The action of NO is mediated by activation of a soluble guanylate cyclase in adjacent vascular smooth muscle cells, thereby increasing intracellular levels of its second messenger, cGMP (128, 129).

It has been shown that iNOS has been implicated in many human diseases associated with inflammation *via* the activation of the c-JNK, p42/44 MAPK, and p38 kinase pathways (130, 131). This isoform of NOS is responsible for the generation of excessive amounts of NO, which leads to tissue injury due to exaggerated generation of oxidative radicals such peroxynitrate. For instance, iNOS is overexpressed in the venous endothelial cells harvested from patients with decompensation CHF (132). Likewise, excessive NO in the heart leads to myocardial depression and reduced contractility in patients and experimental animals with CHF (133).

These findings lead to the “cytokine hypothesis,” which suggests that cytokines play an important pathogenic role in development of HF. This notion is further supported by two studies demonstrating that iNOS knockout mice display less cardiac dysfunction after myocardial infarction than wild-type controls (134). Interestingly, there is negative interplay between iNOS and ANP, where the latter *via* cGMP production increases intracellular calcium levels in murine macrophages resulting in decreased iNOS expression (135).

## Summary and Conclusion

In summary, inflammation and neurohormonal systems appear to interplay one with each other leading to worsening cardiac, pulmonary, and renal functions, which negatively affect patients’ outcome. While the adverse role of the RAAS, SNS, and ET-1 in the pathogenesis of CHF is well established, the involvement of the innate and adaptive immune in the cardiac, renal, and pulmonary manifestations of CHF is still evolving. Although we have shown that the inflammatory system plays a substantial direct and indirect role in heart failure, this observation does not prove unequivocally a causal role in CHF. Therefore, another possibility should be considered, such as that these cytokines are elevated in heart failure in response to the underlying injury, and that they may serve as markers rather than drivers of the disease process. The therapeutic interventions aimed at reducing the activation of the immune cells or blockade of certain cytokines were unsatisfactory. Milestones studies that investigated the effect of TNF-α antagonists, etanercept (136), and infliximab (137) on the composite clinical outcomes of mortality and worsening heart failure in the range of several weeks, in patients with chronic systolic heart failure failed to show any benefit. Infliximab failed to show beneficial effect; this was related possibly to the short term treatment. The RENEWAL investigators suggested several possibilities for the lack of benefit of etanercept; among them, lower investigated doses of etanercept, cytokines may not play an important role in heart failure or alternatively, there is a need to simultaneously target several inflammatory mediators and the predisposition for infection due to etanercept. A recent review of these studies argued that the unfavorable outcomes might be attributed to the population cohorts that were mostly severe with advanced heart failure, toxicity of the treatment, and genetic polymorphism (138). Recently, it was shown in a small cohort of 30 patients with systolic heart failure and acute decompensation; the administration of IL-1 blocker, anakinra reduced the inflammatory burden as shown by reduced C-reactive protein levels within 72 h. However, this study did not address clinical outcomes (139). Cavalli et al. have reported that in a patient who developed fulminant myocarditis with biventricular failure and cardiogenic shock, the administration of anakinra restored cardiac function with clinical improvement (140). Yet, we should await more well-designed studies that may prove to be beneficial in reducing target organ damage and preventing congestion characterizing heart failure.

In light of the limited therapeutic tools of congestion of pulmonary and cardiac etiologies, anti-inflammatory treatment strategies may turn to be a novel approach with promising prognostic consequences in the CRS. Yet, further research is required to understand in depth the interaction between the classic neurohormonal systems and the inflammatory ones, the sources of the latter in the CRS, and their effect on the specific mediators of salt and water transporters at the pulmonary and renal tissues.

## Author Contributions

ZSA and ZA conceived and designed the manuscript structure. ZSA, SK, FB, RI-B, EK, and ZA contributed to writing and reviewing the paper. SK, ZSA, and ZA contributed in preparing the figures.

## Conflict of Interest Statement

The authors declare that the research was conducted in the absence of any commercial or financial relationships that could be construed as a potential conflict of interest.
